# Cloud Computing-Based TagSNP Selection Algorithm for Human Genome Data

**DOI:** 10.3390/ijms16011096

**Published:** 2015-01-05

**Authors:** Che-Lun Hung, Wen-Pei Chen, Guan-Jie Hua, Huiru Zheng, Suh-Jen Jane Tsai, Yaw-Ling Lin

**Affiliations:** 1 Department of Computer Science and Communication Engineering, Providence University, Taichung 43301, Taiwan; 2 Department of Applied Chemistry, Providence University, Taiwan 43301, Taiwan; E-Mails: g1016008@pu.edu.tw (W.-P.C.); sjtsai@pu.edu.tw (S.-J.J.T.); 3 Department of Computer Science, National Tsing Hua University, Hsinchu 30013, Taiwan; E-Mail: gt758215@gmail.com; 4 School of Computing and Mathematics, University of Ulster, Newtownabbey BT37 0QB, UK; E-Mail: h.zheng@ulster.ac.uk; 5 Department of Computer Science and Information Engineering, Providence University, Taichung 43301, Taiwan

**Keywords:** SNPs, haplotype, cloud computing, parallel processing, MapReduce

## Abstract

Single nucleotide polymorphisms (SNPs) play a fundamental role in human genetic variation and are used in medical diagnostics, phylogeny construction, and drug design. They provide the highest-resolution genetic fingerprint for identifying disease associations and human features. Haplotypes are regions of linked genetic variants that are closely spaced on the genome and tend to be inherited together. Genetics research has revealed SNPs within certain haplotype blocks that introduce few distinct common haplotypes into most of the population. Haplotype block structures are used in association-based methods to map disease genes. In this paper, we propose an efficient algorithm for identifying haplotype blocks in the genome. In chromosomal haplotype data retrieved from the HapMap project website, the proposed algorithm identified longer haplotype blocks than an existing algorithm. To enhance its performance, we extended the proposed algorithm into a parallel algorithm that copies data in parallel via the Hadoop MapReduce framework. The proposed MapReduce-paralleled combinatorial algorithm performed well on real-world data obtained from the HapMap dataset; the improvement in computational efficiency was proportional to the number of processors used.

## 1. Introduction

Genome-wide association studies based on linkage disequilibrium (LD) offer a promising approach for detecting the genetic variations underlying common human diseases. Single nucleotide polymorphisms (SNPs) are useful markers in disease association research because they are abundant along the human genome, mutate at low rates, and are accessible to high-throughput genotyping. SNP refers to the existence of two specific nucleotides at a single locus in a population. A haplotype can be regarded as part of SNP on a single chromosome. Throughout the last decade, haplotype analysis has identified DNA variations relevant to several common and complex diseases [1,2,3,4,5,6]. According to many studies, the human genome may be structured into haplotype blocks, and most haplotype structures are obtained from only a small number of SNPs called tagSNPs [7,8,9,10,11,12,13].

Block structures can be defined in several ways. Four main criteria for haplotype block partitioning are haplotype diversity, LD, the four-gamete test, and information complexity. In diversity-based methods [9,14,15], a block is defined as a region in which a certain percentage of haplotypes, called common haplotypes, are present in more than a certain percentage of the population. In LD-based methods [8,16], blocks comprise regions of high pair-wise LD separated by regions of low pair-wise LD. Methods based on the four-gamete test [17,18] define a block as a recombination-free region in consecutive SNPs. Anderson *et al.* [19] developed an information complexity-based method that finds the block boundaries in statistical-model selection. They applied the minimum description length (MDL) criterion to select the block designations that configure the structure within the data.

Diversity-based methods can be categorized into two groups. In the first group, strings of SNPs are divided into blocks based on the LD decay across block boundaries; in the second group, blocks are delineated by some haplotype-diversity measure within the blocks. Patil *et al.* [9] defined a haplotype block as a region that represents either a certain percentage of all observed haplotypes at least *n* times or a given threshold in the sample. Applying the optimization criteria outlined by Zhang *et al.* [12,20], they described a general algorithm that defines block boundaries in a way that minimizes the number of SNPs required to identify all haplotypes in a region. Patil *et al.* [9] defined the haplotype structure of human chromosome 21 as 4563 tagSNPs in 4135 blocks. In each block, they stipulated that at least 80% of the haplotype must be represented at least twice.

In this paper, we propose a diversity function for measuring haplotype block quality. We implement the diversity function in programs (FinKLB) that partition the haplotypes into blocks. Our algorithm identifies segmentations of *k* blocks while maximizing the total length of the SNPs. The algorithm is applied to a haplotype dataset downloaded from the HapMap project. Like Zhang *et al.* [12,20], we adopted the criteria of Patil *et al.* [9]; that is, a block must represent at least 80% of the haplotypes more than once. However, our algorithm partitions the haplotypes into fewer blocks than Zhang *et al.*’s algorithm [20]. In our results, the average block length is longer and most of the chromosomal information is captured in a minority of the blocks. More specifically, we capture 70% of the chromosome in 40% of our haplotype blocks. However, when implemented by existing approaches, the calculations are complicated and computationally intensive. To enhance the performance of our algorithm, we enable parallel data copying through the Hadoop MapReduce framework.

Hadoop [21] is a software framework that supports data-intensive distributed applications. It can process petabytes of data via thousands of nodes. Hadoop supports the MapReduce programming model [22], by which applications for parallel processing of large datasets are written in a cloud computing environment. MapReduce enables distributed computing of the map and reduces the number of operations. All map operations are mutually independent, and all maps can perform tasks in parallel. In practice, the total number of maps is limited by the data source and/or the number of CPUs near the data. Similarly, reduce operations are performed by a set of reducers, which receive the outputs of the map operation with the same key after shuffling and sorting. Importantly, by distributing the developed computing applications through Hadoop, we improve the fault tolerance of the applications. If the running nodes or network components in a large cluster fail during a job execution, Hadoop can guide the jobs toward successful completion. Bioinformatics applications are notoriously time-intensive, and jobs may require weeks or months to complete. Traditional parallel models such as MPI, OpenMP, and multi-thread are unsuited to such applications, because a local fault may cause the entire application to fail. Moreover, in the MPI model, the master node sends the data to slave nodes for computation. This network structure may create a performance bottleneck during real-world large-data processing, which is avoided by the Hadoop platform. Recently, Hadoop has been applied in various bioinformatics domains [23,24,25,26].

In this paper, we implement a parallel diversity-based haplotype block selection algorithm on the Hadoop MapReduce framework. The mapper calculates the required diversity and tagSNPs in each block, while the reducer locates the blocks. Experimental results indicate that the proposed algorithm is significantly faster than the corresponding sequential algorithms as the number of map operations increases.

## 2. Results and Discussion

All of the experiments were performed on three IBM blade servers in our cloud computation laboratory. Each server is equipped with two Quad-Core Intel Xeon 2.26-GHz CPUs, 24-GB RAM, and a 296-GB hard disk running under the operating system Ubuntu (v.10.4) with a Hadoop (v.0.2) MapReduce platform. Under the current system environment, the server execution processes control up to 8 map operations and 8 reduce operations and up to 24 map/reduce operations.

### 2.1. ASW Data Characteristics

We first applied our dynamic programming algorithm to haplotype datasets retrieved from the HapMap project. The datasets include chromosomes 7, 8, 9, and 10 from individuals of African ancestry in the Southwest USA (abbreviated ASW). Each dataset contains 26 individuals and 75,320; 75,272; 63,612; and 73,832 SNPs, respectively. Table 1 compares the results of our algorithm (FinKLB) with those of Zhang *et al.* [20] under the criterion of 80% common haplotype coverage. Zhang’s algorithm partitions haplotype blocks while minimizing the number of tagSNPs; in contrast, we partition haplotypes into a minimum number of blocks. In all cases, Zhang’s algorithm yielded fewer tagSNPs, while FinKLB generated longer average block lengths. On average, our algorithm reduces the haplotype block number by 5% while increasing the number of tagSNPs by 11%. In this experiment, both algorithms were executed on a single CPU (Intel Xeon 2.26 GHz). The proposed algorithm can run several hundred times faster than Zhang’s algorithm, by virtue of its efficient tagSNP selection method.

**Figure 1:**
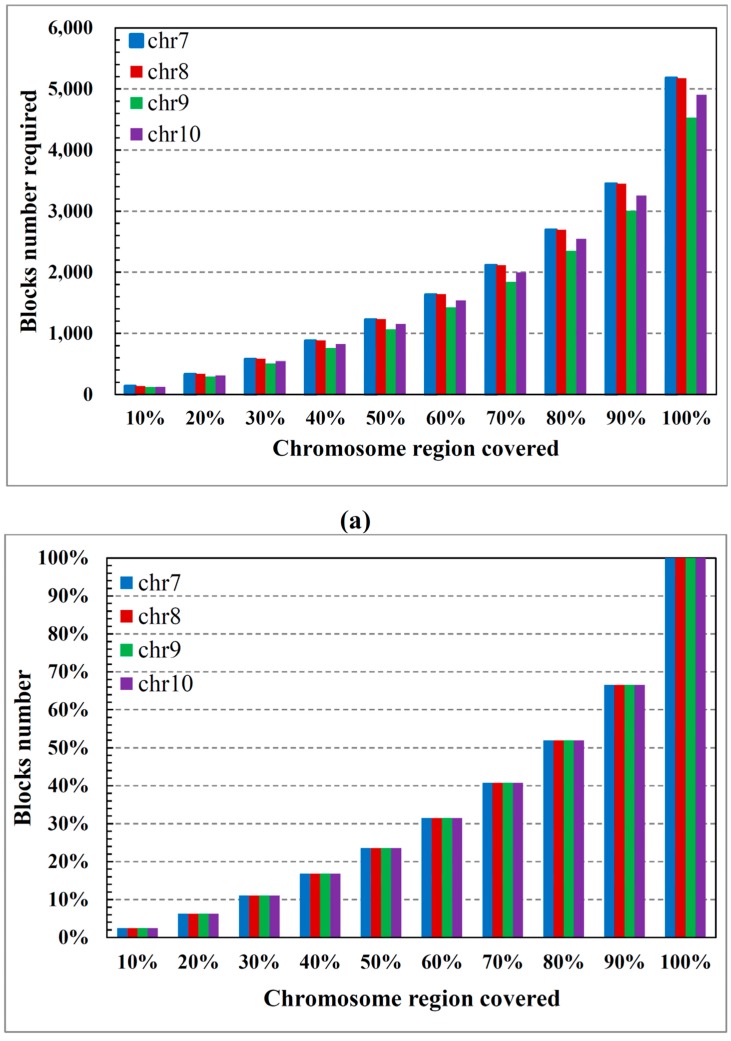
Blocks required to cover 10% increments of the chromosomal region: (**a**) number of blocks and (**b**) percentage of blocks.

**Figure 2:**
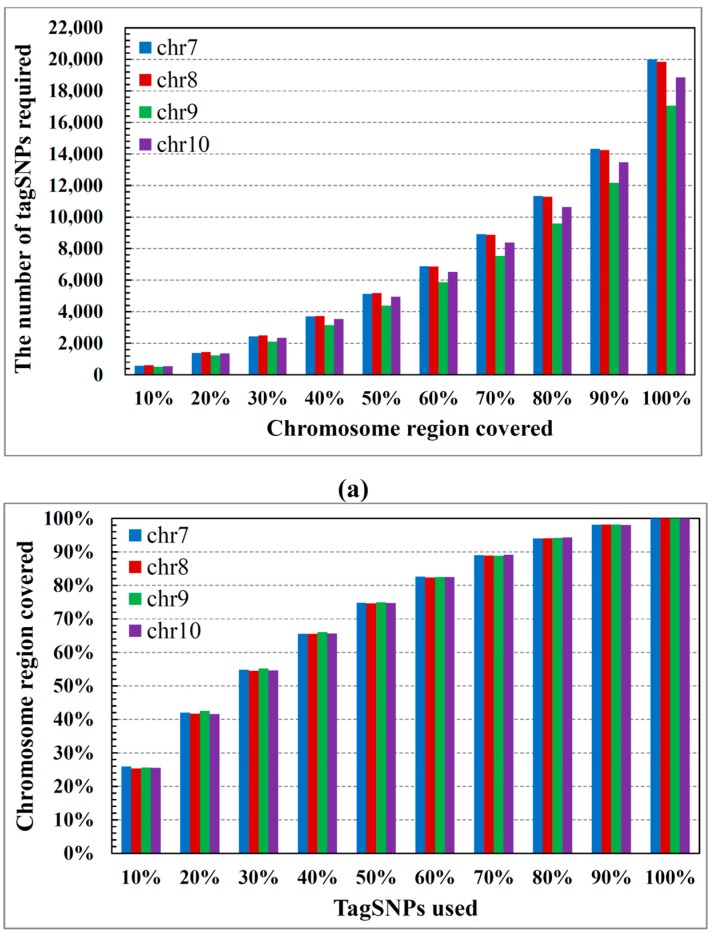
TagSNPs required to cover 10% increments of the chromosomal region: (**a**) number of TagSNPs and (**b**) percentage of tagSNPs.

**Figure 3:**
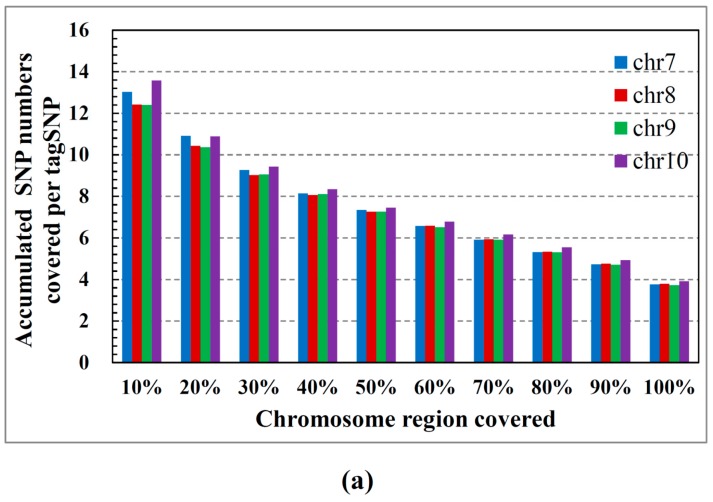

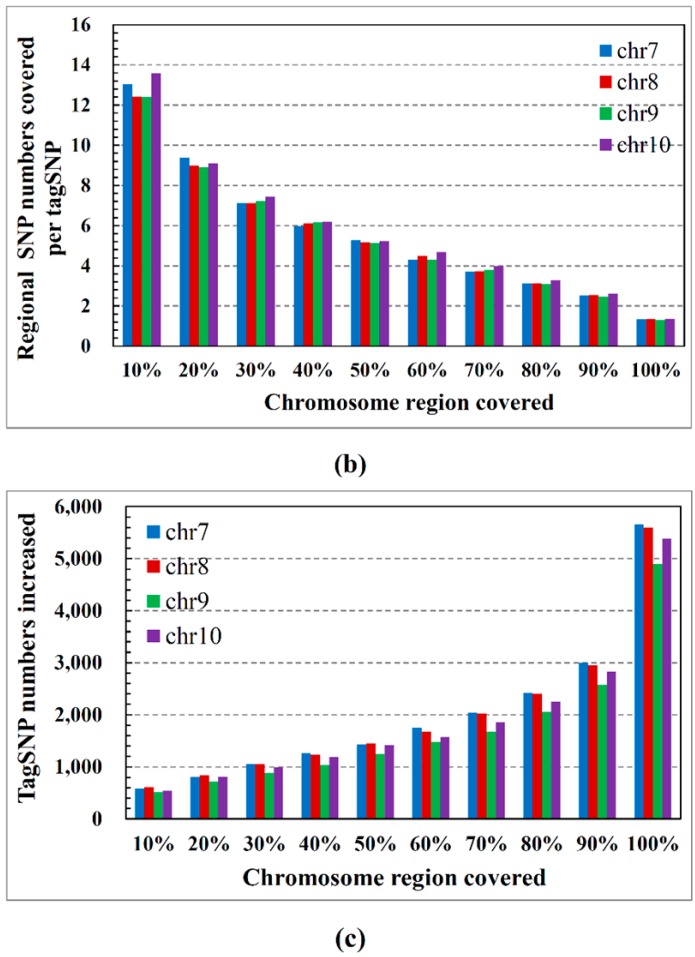
(**a**) Average number of SNPs covered by each tagSNP; (**b**) SNP numbers covered per tagSNP, for each 10% coverage of the chromosomal region and (**c**) Increase in the number of tagSNPs required to cover each 10% increment of the chromosomal region.

Figure 1a relates the block number to the percentage of the chromosomal region (common SNPs) covered by the total block. Note that a wide region of the chromosome is covered by only a few blocks. More specifically, in all cases, approximately 40% of the blocks (see Figure 1b) cover 70% of the chromosomal region. Figure 2a shows the number of tagSNPs required for the blocks to cover a certain percentage of the chromosomal region. According to this figure, 8000 tagSNPs are sufficient for a 70% coverage of the genome (less than 50% of the tagSNPs required in Figure 2b). This coverage captures most of the haplotype information, confirming that our method embodies most of the regional chromosome information in just a few tagSNPs. Figure 3a shows the percentage of common SNPs covered by each tagSNP on average, *versus* the percentage of the chromosomal region covered by the blocks. Note that as more of the chromosomal region is covered by the blocks, fewer common SNPs are covered by each tagSNP (on average). Figure 3b shows the number of SNPs covered per tagSNP for each 10% coverage of the chromosomal region. Interestingly, the marginal utility of tagSNPs decreases with increasing genome coverage. Figure 3c relates the percentage coverage of the chromosomal region to the number of tagSNPs required for each coverage.

**Table 1:** The properties of haplotype blocks obtained by Zhang *et al.*’s algorithm [20] and our FinKLB algorithm. The datasets contain chromosomes 7, 8, 9, and 10 from an ASW population. The criterion was 80% coverage of the common haplotype.

Common SNPs/Block	Zhang					FinKLB				
	No. of Blocks	Length	Avg. Length	All Blocks (%)	Common SNPS (%)	No. of Blocks	Length	Avg. Length	All Blocks (%)	Common SNPs (%)
**ASW_chr7**										
*<* 15	3525	28,090	7.97	64.29	37.29	3250	29,822	9.18	62.82	39.59
15 to 30	1604	32,524	20.28	29.25	43.18	1603	32,138	20.05	30.98	42.67
*>* 30	354	14,706	41.54	6.46	19.53	321	13,360	41.62	6.20	17.74
Total	5483	75,320	13.74	100.00	100.00	5174	75,320	14.56	100.00	100.00
Max. Blocks	102					107				
Tag SNP	18,012					19,990				
CPU Time	409,834(s) ≡ 113.83(h)					783(s) ≡ 0.22(h)				
**ASW_chr8**										
*<* 15	3514	27,976	7.96	63.90	37.17	3225	29,837	9.25	62.32	39.64
15 to 30	1640	33,156	20.22	29.82	44.05	1638	32,798	20.02	31.65	43.57
*>* 30	345	14,140	40.99	6.28	18.78	312	12,637	40.50	6.03	16.79
Total	5499	75,272	13.69	100	100.00	5175	75,272	14.55	100.00	100.00
Max. Blocks	105					105				
Tag SNP	17,957					19,844				
CPU Time	299,970(s) ≡ 83.32(h)					924(s) ≡ 0.25(h)				
**ASW_chr9**										
*<* 15	3175	24,607	7.75	65.98	38.68	2945	26,714	9.07	65.02	42.00
15 to 30	1343	27,093	20.17	27.91	42.59	1330	26,664	20.05	29.37	41.92
*>* 30	294	11,912	40.52	6.11	18.73	254	10,234	40.29	5.61	16.09
Total	4812	63,612	13.22	100.00	100.00	4529	63,612	14.05	100	100.00
Max. Blocks	83					83				
Tag SNP	15,308					17,064				
CPU Time	47,786(s) ≡ 13.27(h)					645(s) ≡ 0.17(h)				
**ASW_chr10**										
*<* 15	3261	25,850	7.93	62.52	35.01	2973	27,623	9.29	60.62	37.41
15 to 30	1556	31,559	20.28	29.83	42.75	1585	31,855	20.10	32.31	43.15
*>* 30	399	16,423	41.16	7.65	22.24	347	14,354	41.37	7.07	19.44
Total	5216	73,832	14.15	100	100.00	4905	73,832	15.05	100	100.00
Max. Blocks	112					112				
Tag SNP	17,012					18,862				
CPU Time	46,580(s) ≡ 12.93(h)					919(s) ≡ 0.25(h)				

### 2.2. Performance on Cloud Computing

In the second experiment, we evaluated and compared the performance of our Hadoop-based dynamic algorithm executed on a single CPU and launched onto various mappers. The SNP haplotype data were gathered from the International HapMap Project [27], a multi-country effort to identify and catalog the genetic similarities and differences among human beings. This project collects the genetic sequences of numerous diverse individuals. We downloaded the sequence data (Chromosome 1) from the HapMap3 Genome Browser release #2, collected from individuals of African ancestry in the Southwest USA (ASW). ASW includes 136 Chromosome 1 (chr 1) sequences (patterns) and contains 116,416 SNPs. These sequences provide the input data for our experiments. The diversity scores of the blocks were computed by Equation (1).

*δ_s_*(B) = 1 – *C*/*U* = *S*/*U* (1)

where *U*, *C*, and *S* denote the number of unambiguous, common, and singleton haplotypes, respectively.

**Figure 4:**
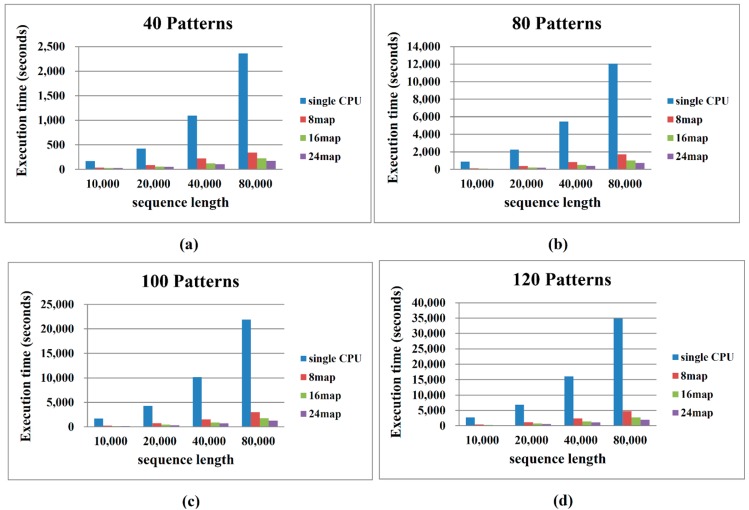
Performance comparison between sequential and MapReduce haplotype block selection (block size = 300 bp). (**a**) Number of Patterns is 40; (**b**) Number of Patterns is 80; (**c**) Number of Patterns is 100 and (**d**) Number of Patterns is 120.

**Figure 5:**
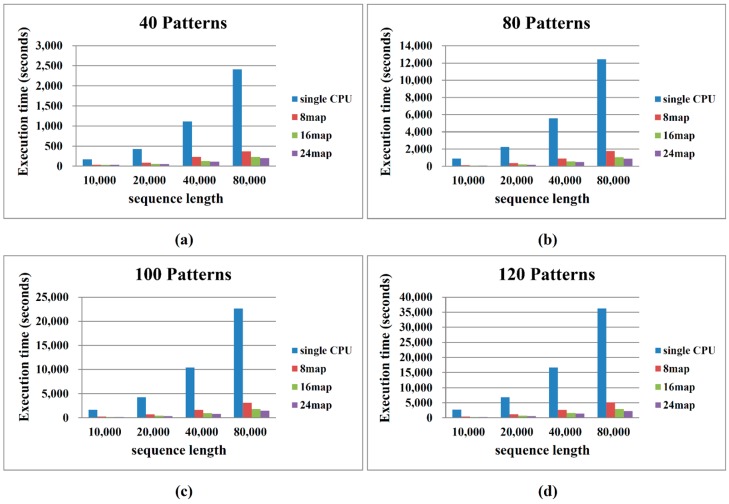
Performance comparison between sequential and MapReduce haplotype block selection (block size = 500 bp). (**a**) Number of Patterns is 40; (**b**) Number of Patterns is 80; (**c**) Number of Patterns is 100 and (**d**) Number of Patterns is 120.

To assess the performance of the proposed Hadoop MapReduce algorithm, we compared the computational time required to process various sequence data and different numbers of map/reduce operations. The performances of both the sequential and the proposed algorithm depend on the number and length of the patterns. Patil *et al.* [9] proposed that haplotype blocks reside within 300-bp and 500-bp regions. Therefore, we assumed block sizes of 300 bp and 500 bp. The diversity scores are based on the corresponding block sizes and are denoted as {*δ*(1, 1), *δ*(1, 2), …, *δ*(1, 500), *δ*(2, 2), …, *δ*(2, 501), *δ*(3, 3), …, *δ*(*L*, *L*)}. Figure 4 and Figure 5 compare the performances of the sequential algorithm and our MapReduce framework-based algorithm for block sizes of 300 bp and 500 bp, respectively. The computational time increases with increasing pattern number and sequence length. Our algorithm processes the 300-bp block more rapidly than the 500-bp block. More patterns and longer sequence lengths incur a higher computational cost. These results are consistent with the algorithm analysis presented in the previous section.

**Figure 6:**
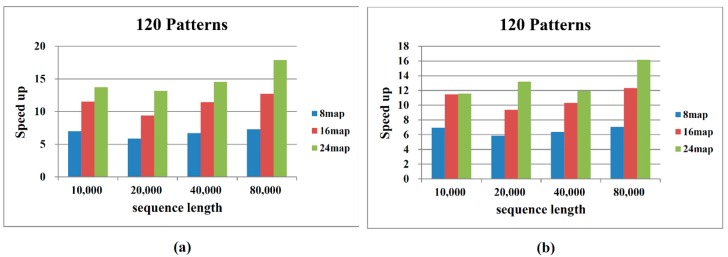
Speed-up comparison between sequential and MapReduce haplotype block selection: (**a**) block size of 300 bp and (**b**) block size of 500 bp.

Deploying more map operations effectively reduces the computational time. Deployment of 8 and 16 map operations improves the computation time by more than sixfold and tenfold, respectively, with respect to implementation on a single CPU. When the number of map operations is increased to 24, moderate enhancements are observed for smaller sequence lengths (10,000–40,000 bp), since 16 and 24 operations split the dataset into similar sizes. As evident in Figure 4, Figure 5 and Figure 6, the computation efficiency of our algorithm is proportional to the number of processors employed.

## 3. Methods

SNPs are chromosomal positions at which two (or more) specific nucleotides are observed in at least 10% of the population [9]. The nucleotides within SNP are called alleles. The present paper is restricted to *biallelic* SNPs, which have only two different alleles, and constitute the vast majority of SNPs.

### 3.1. Diversity Function

The input to the haplotype blocking problem is a set of *m* haplotype vectors. Each position in each vector is associated with a site of interest on the chromosome. Usually, the *major* and *minor* alleles in the haplotype vector are assigned the values of 0 and 1, respectively.

**Figure 7:**
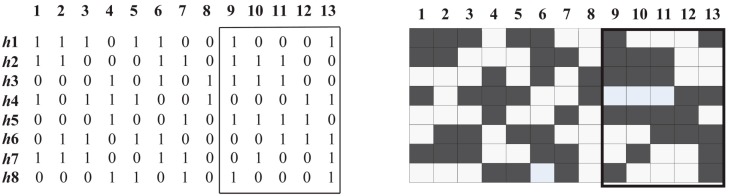
A haplotype matrix *B* and its corresponding submatrix *M*(8, 13).

Let the haplotype matrix *A* be an *m* × *n* matrix of *m* observations over *n* markers (sites). We denote the *j*-th allele of observation *i* by *A_ij_*. For the sake of simplicity, we assume that *A_ij_* Ȇ {0, 1}. A block, or marker interval, [*j*, *k*] = {*j*, *j* + 1, …, *k*} is defined by two marker indices 1 ≤ *j* ≤ *k* ≤ *n*. A *segmentation* is a set of non-overlapping non-empty marker intervals and is full if the union of the intervals is [1, *n*]. The data matrix within interval [*j*, *k*] is denoted by *M*(*j*, *k*); the values of the *i*-th observation are denoted by *M*(*i*, *j*, *k*), a binary string of length *k* − *j* + 1. As an example, an 8 × 13 haplotype matrix is presented in Figure 7.

Within an interval [*j*, *k*], the *diversity function δ*: [*j*, *k*] → [*0*, *1*] quantifies the diversity of the submatrix *M*(*j*, *k*). If *j* ≤ *j*' and *k*' ≤ *k*, then the interval [*j*', *k*'] is a subinterval of [*j*, *k*], written as [*j*', *k*'] Ȅ [*j*, *k*]. Note that the *δ*-function is a monotonically non-decreasing function from [1…*n*, 1…*n*] to the unit real interval [*0*, *1*]; that is, 0 ≤ (*j*', *k*') ≤ (*j*, *k*) ≤ 1 whenever [*j*', *k*'] Ȅ [*j*, *k*].

Given an input set of *n* haplotype vectors, the *haplotype block selection* (*HBS*) problem returns a segmentation of marker intervals, revealing the non-overlapped haplotype blocks of interest in the chromosome.

### 3.2. Common Haplotypes

Two haplotypes are said to be *compatible* if the alleles are identical at all loci for which no data are missing; otherwise, the two haplotypes are said to be *incompatible*. Following Patil *et al.* [9], we define ambiguous haplotypes as haplotypes that are compatible with at least two haplotypes that are themselves incompatible. It should be noted that all haplotypes are unambiguous if the data are complete. Haplotypes represented more than once in a block are called common haplotypes, whereas those incompatible with any others are called *singleton* haplotypes.

We are mainly interested in the common haplotypes. Therefore, we require a significant fraction of common haplotypes in the final block partition. Patil *et al.* [9] stipulated that at least 80% of the unambiguous haplotypes appear more than once; that is, *α =* 80%, where *α* is the *coverage* of common haplotypes in a block (excluding the ambiguous haplotypes). The coverage of block *B* can be mathematically formulated as a diversity measure: Equation (1). For example, the submatrix *M*(9, 13) of Figure 7 can be viewed as a sample *S* = {10001, 11100, 00011, 11110, 00001, 01001}. It follows that *δ*(*S*) = *δ*(*M*(9, 13)) =1 − 4/8 = 4/8.

### 3.3. Haplotype Block Partitioning

Given a haplotype matrix *A* and a diversity upper limit *D*, we wish to find *k* disjoint blocks with diversity less than *D* while maximizing the total length. That is, we output the set *S* = {*B*_1_, *B*_2_, …, *B*_k_} with *δ_S_*(*B*) ≤ *D* for each *B* Ȇ *S*, such that |*B*_1_| + |*B*_2_| + … + |*B*_k_| is maximized. Here |*B*_i_| denotes the length of block *B*_i_. Assuming a monotonic diversity function, we preprocess the given haplotype matrix to find the indices of the site farthest from the current site, called the *good partner site*. We then solve the longest-*k*-block problem by a dynamic programing algorithm [28]. The good partner of locus *i* refers to the left farthest locus from *i*, *L*[*i*], such that *δ_S_*(*L*[*i*], *i*) ≤ *D*. We define *f*(*k*, *i*, *j*) = max{|*S*|}, where *k* is the number of blocks. If the diversity function *δ_S_* is monotonic, the recurrence function is given by

*f*(*k*, 1, *j*) = max{*f*(*k*, 1, *j* − 1), *f*(*k* − 1, 1, *L*[*j*] − 1) + *j* − *L*[*j*] + 1} (2)

The recurrence relation assumes that either the *k-*th block of the maximal segment *S* in [1, *j*] excludes site *j* or block [*L*[*j*], *j*] is the last block of *S*.

### 3.4. TagSNPs Selection

For each block, we minimize the number of SNPs that uniquely distinguish all common haplotypes in the block. These SNPs, referred to as tagSNPs, can be interpreted as the signature of the haplotype block partition. Since the tagSNPs capture most of the haplotype diversity, they potentially capture most of the information for associating a trait and its marker loci [26].

The sets of haplotypes and SNP sites in a haplotype block are denoted as *H* and *S*, respectively. Obviously, each SNP site *s* Ȇ *S* partitions the haplotypes into two groups *G_1_* and *G_2_*, whose allele at site *s* is major (0) and minor (1), respectively. Site *s* defines a partition π*_s_* = {*G_1_*,*G_2_*} on *H*, *G_i_* Ȅ *H*. These two subsets are disjointed, and their union *H*. From this observation, we can regard the tagSNP selection problem as minimizing the number of SNP sites such that the partitions defined by these sites distinguish all common haplotypes in the block. To this end, we select the tagSNPs in the haplotype blocks by the following strategy: The common haplotypes in a given block are separated into *k* distinct groups, and the smallest number of required SNPs is decided. Finally, adopting our previously proposed tagSNPs selection method [29], we select a loci set *T* containing the minimum number of SNPs such that partition π*_t_* defines *k* equivalence classes. To generate the next candidate tagSNP loci set, the algorithm enumerates the next *γ*-combination in a lexicographic order. The algorithm iterates until each group is uniquely distinguished.

### 3.5. Hadoop Framework

The software framework Hadoop coordinates computing nodes, enabling parallel processing of the distributed data. Hadoop develops parallel computing applications using the map/reduce parallel programming model. The standard map/reduce mechanism is adopted by many successful cloud computing service providers, including Yahoo, Amazon EC2, IBM, and Google. An application developed by MapReduce comprises a map stage and an optional reduce stage. The input data are split into smaller chunks corresponding to the number of maps. The map stage outputs <*key*, *value*> pairs from all map nodes, which are classified by *key* before being distributed to the reduce stage. The reduce stage combines *value* and *key* and outputs <*key*, *value*> pairs, each with a unique *key* value.

The Hadoop cluster comprises a single master and multiple slave nodes. The master node consists of a *jobtracker*, *tasktracker*, *namenode*, and *datanode*. The slave nodes (which perform the computations) consist of a datanode and tasktracker. The jobtracker service farms out the MapReduce tasks to specific nodes in the cluster, ideally the nodes holding the data, or at least within the same rack. Tasks (Map, Reduce, and Shuffle operations) allocated by jobtrackers are accepted by tasktrackers.

Hadoop distributed file system (HDFS) is the primary file system used by the Hadoop framework. Each input file is split into data blocks that are distributed to datanodes. Hadoop also creates multiple replicates of data blocks, which are distributed to datanodes throughout the cluster to enable reliable, extremely rapid computations. The namenode serves as both a directory namespace manager and a node metadata manager for the HDFS. A single namenode runs in the HDFS architecture.

### 3.6. Hadoop-Based Block Partitioning and Selection Scheme

**Figure 8:**
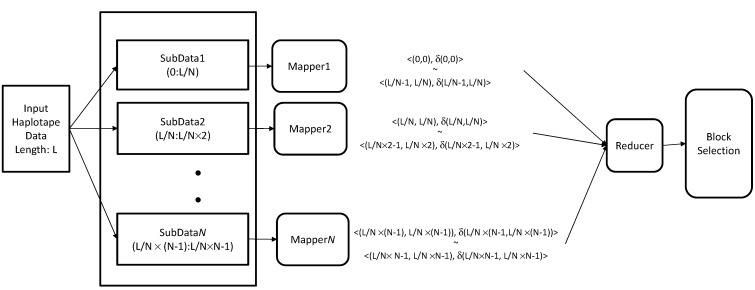
Haplotype block partitioning and selection based on the MapReduce framework.

Figure 8 illustrates the use of the MapReduce framework in the block partitioning and selection scheme. Assuming *N* map operations and a pattern length of *L*, we split the input *N × L* haplotype matrix into *L/N* chunks. Each map calculates the diversity scores of each block within its allocated data chunk. Thus, the <*key*, *value*> pairs of each map are output as <(*block start number*, *block end number*), *diversity score*> pairs. Further, *map_i_* calculates the diversity scores of the blocks {*δ*(*i*∙*N*/*L*, *i*∙*N*/*L*), *δ*(*i*∙*N*/*L*, *i*∙*N*/*L* + 1), …, *δ*(*i*∙*N*/*L* + *N*/*L*, *i*∙*N*/*L* + *N*/*L*)}.

Therefore, (*N*/*L*)^2^ diversity scores are computed for each map. The reduce stage executes the haplotype block selection algorithm. Since the selection algorithm is linear in time, parallel computation is not required, and a single reduce operation is sufficient. The haplotype block selection algorithm was described in Subsection 3.3. The reduce operation finds the longest block by merging blocks with interesting diversity scores.

## 4. Conclusions

By investigating SNPs and haplotype blocks, biomedical researchers can detect inheritable diseases and contribute to human race classification and evolutionary research. In this study, we developed a haplotype block-partition system based on our dynamic programming method that maximizes the total block length. Given an appropriate diversity function, the block selection problem can be viewed as segmenting the haplotype matrix such that the diversities of the selected blocks satisfy a given constraint. In haplotype data extracted from HapMap, our method identified longer and fewer blocks (a number reduction of 5%) than an existing algorithm. Our method revealed that only a few blocks are sufficient to cover a wide range of the genome and that a few tagSNPs capture most of the local genomic information.

Rather than genotype all SNP markers on the chromosome, the required information can be obtained from the genotype information on the tagSNPs. Approximately 50% of the SNPs can account for more than 70% of the common haplotypes on each chromosome. Thus, studying the tagSNPs can significantly enhance the performance of genotyping, without a significant loss of the haplotype information. Because the result of block partitioning and the meaning of each haplotype block depend on the measurement formula, we measured the block quality by using a diversity function. We also provided an efficient algorithm that selects tagSNPs within a haplotype block. Traditionally, haplotype blocks are detected by time-consuming dynamic programming approaches. As bioinformatic data accumulate, these sequential methods require imminent assistance from emerging parallel processing methodologies. In this paper, we discussed the development of our parallelized framework and demonstrated its benefit to our original dynamic programming algorithms. The Hadoop MapReduce framework re-submits jobs to other nodes if the working node fails. Therefore, Hadoop can process large amounts of sequence data without the risk of stoppage by node failure. Finally, we compared the performance of our algorithm by varying the sequence length, number of patterns, and block size. According to the experimental results, our proposed algorithm significantly decreases the computational cost of sequence data processing.
